# Associations between Motives for Physical Exercise, Body Composition and Cardiorespiratory Fitness: A Cross-Sectional Study

**DOI:** 10.3390/ijerph192114128

**Published:** 2022-10-29

**Authors:** Pablo Galan-Lopez, Isabel Lopez-Cobo, Irene García-Lázaro, Francis Ries

**Affiliations:** 1 Department of Communication and Education, Faculty of Social and Human Sciences, Universidad Loyola Andalucía, 41704 Seville, Spain; 2 Department of Didactics and Educational Organization, University of Seville, 41013 Seville, Spain; 3 Department of Physical Education and Sports, Faculty of Educational Sciences, University of Seville, 41013 Seville, Spain

**Keywords:** physical exercise, motivation, cardiorespiratory fitness, quantitative research, late childhood, adolescents’ health

## Abstract

Adolescents’ need for some minimum amount of daily physical exercise has been widely studied so as to assist better health outcomes and to reduce future obesity rates. However, the motivations of adolescents to exercise are less well-known. This manuscript aims to analyze the motives that explain the practice of physical exercise in adolescents and the possible associations with elements of body composition and cardiorespiratory fitness. For this purpose, the Self-Report of Motives for the Practice of Physical Exercise questionnaire (AMPEF) was administered to 917 students between 13–16 years of age (50.1% girls, 49.9% boys, M age = 14.82) from Seville, Spain. Subscales Ill-Health Avoidance and Positive Health, Revitalization and Enjoyment, Strength and Endurance, and Challenge represent the participants’ main reasons for practicing physical exercise. Associations between BMI and FAT % with the subscales Weight Management and Appearance (direct association) and Revitalization and Enjoyment (inverse association) were found. A direct association between cardiorespiratory fitness and Revitalization and Enjoyment, Competition, Strength, and Endurance and Challenge subscales was found for both genders. Conclusions emphasize the practice of physical exercise in adolescents due to intrinsic motives based on improving their state of health, increasing their levels of strength and endurance (boys), and achieving short-term objectives (girls).

## 1. Introduction

Physical exercise (PE) is a fundamental part of a healthy lifestyle and has been widely associated with health benefits. High levels of sedentary lifestyle and physical inactivity have increased the prevalence of pathologies that affect today’s society [1,2]. Regarding the benefits of PE practice, the literature is extensive. It is considered a vital tool for increasing and maintaining a good quality of life, especially in pandemic and post-pandemic times [3]. It is recommended that young people perform at least 60 min of moderate to vigorous exercise every day per week [4,5]. On the other hand, the lack of PE in adolescents interferes with the attainment of benefits. [6,7]. This situation underlines the need to dedicate more attention to the determinants of the development of PE (i.e., the intention to be physically active, the perceived health benefits, motivation toward PE, self-efficacy perception, support, influence from significant others to practice PE, and the availability of sports facilities). 

Overweight status and obesity are increasingly prevalent among children and adolescents in developed countries [8]. In Spain, the prevalence of overweight and obesity exceeds 35% in adolescents: Spain continues to have the second highest childhood overweight and obesity prevalence in Europe [9]. In addition, the consequences of this prevalence affect immediate health and, in the long-term, create risk profiles in suffering diseases within adulthood [10,11,12]. During adolescence, health risk behaviors are frequently adopted [13,14], observing, in some cases, concurrence between them [15]. For instance, PE and physical fitness levels are directly related to parameters associated with health [16], among them the body mass index (BMI) in terms of percentages [17]. The regular practice of PE and adequate cardiorespiratory fitness (CRF) are related to a low cardiovascular risk profile and are strong indicators of coronary health [18,19]. Furthermore, although cardiovascular disease (CVD) events frequently appear after fifty, their precursors seem to have their origin in childhood and adolescence [20].

A concern about motives for practicing PE is shown in many studies where its importance is highlighted during adolescence due to the significant decrease of PE levels between the ages of 12 to 16 years [21,22,23,24,25]. Consequently, there has been an increased interest based on motivational theories to develop and conduct interventions to promote PE in adolescents [26,27]. The motivational aspects influencing adolescents’ sports practice fluctuate from improving physical appearance to well-being through to fun and social relationships. In addition, ego-orientation and achievement goals are relevant as they influence decision-making regarding participation in recreational or competitive sports activities [28]. Therefore, establishing healthy habits is fundamental for adolescents’ physical and psychological development [29,30]. 

The multiple factors involved in performing PE in adolescence need to be studied and special focus should be given to the motives of PE practice and their relationships with specific physical parameters. Notwithstanding all the background mentioned above, there is insufficient evidence relating adolescents’ motives for practicing PE to variables of body composition and cardiorespiratory fitness. Thus, the objective of this study was to analyze the motives that explain the practice of physical exercise in adolescents, and possible associations with elements of body composition and cardiorespiratory fitness.

## 2. Materials and Methods

This research follows the quantitative approach. This proposal uses a cross-sectional and descriptive design according to the standards proposed by the Declaration of Helsinki (last updated in 2000). This study follows the guidelines proposed by the Good Clinical Practice of the European Medicine Agency. Moreover, the research was approved by the Bioethical Committee of Junta de Andalucía (regional government) (Ref.: 0310-N-17). 

### 2.1. Participants

This study involves teenagers from 13 to 16 years old. The participants belong to five different public and charter-schools in Seville (Spain). A confidence interval of 95% with a 10% margin of error was estimated in the sample selection, which was developed by convenience sampling [31]. Nine hundred and ninety-one teenagers (491 boys and 500 girls) represent the population. Nine hundred and seventeen adolescents (458 boys (49.9%), Mage = 14.82, SD = 1.11; 459 girls (50.1%), Mage = 14.83, SD = 1.09) were the final participants which makes for a 92.53% participation ratio. Measurements were collected during three months of physical education classes during the academic year 2017–2018.

### 2.2. Instruments

#### 2.2.1. Autoinforme de Motivos para la Práctica de Ejercicio Físico: AMPEF

The “Auto-Informe de Motivos para la Práctica de Ejercicio Físico” (AMPEF, Self-Report on Motivation for Exercising) was the tool used to measure motives in the practice of exercise. This instrument is an adaptation of Capdevila, Niñerola, and Pintanel’s [32] contribution which, in turn, is based on the Exercise Motivations Inventory-2 (EMI-2) [33], and is validated for adolescent populations. The AMPEF scale presents 48 items divided in 11 subscales: Weight Management and Appearance, Revitalization and Enjoyment, Ill-Health Avoidance and Positive Health, Competition, Affiliation, Strength and Endurance, Social Recognition, Stress Management, Nimbleness, Challenge, and Health Pressures. The instrument presents a Likert scale from 0 (nothing true for me) to 10 (totally true for me).

#### 2.2.2. Body Composition

Some body composition parameters, such as height, weight, waist circumference, BMI, and FAT % were selected to be measured through a different version of the ALPHA-Fitness Battery (ref: 2006120). Some aspects were not considered (i.e., skin folds) since time did not allow the authors to conduct these measurements. However, body fat percentage was considered through bioelectrical impedance (Tanita Inner Scan BF-689, Tanita, Tokyo, Japan), validated by the FDA. The authors also considered the ALPHA-Fitness Battery to be suitable guidelines in selecting the measures [34].

### 2.3. Data Analysis

Data are presented as statistical values, specifically mean (M) and standard deviation (SD). 

Normality in the variables’ distribution was verified through Kolmogorov-Smirnoff test. A parametric test (*T* Student) is conducted in the different cases to compare body composition variables with diverse motivation subscales to examine women and men’ responses in different motivation subscales. Statistical information was obtained through descriptive analyses (i.e., mean, standard deviation, frequency presented in tables and scatter plots). Moreover, reliability was tested and an ANOVA test was conducted to study the existence of gender differences. Additionally, a post hoc Bonferroni test was conducted in case significant differences existed. The authors established the parameter of significance as *p* < 0.05. IBM SPSS Statistics v.24 (Chicago, IL, USA) was used to conduct the analyses.

## 3. Results

Table 1 shows the values of the age and body composition variables for the whole sample by gender. During the analysis of the anthropometric characteristics, although the boys presented statistically higher differences in the weight and height variables, there were no statistically significant differences in BMI. Moreover, it was verified that the boys presented a lower body fat percentage but a significantly higher waist perimeter. As to cardiorespiratory fitness, it was confirmed that the boys presented substantially higher performance.

Table 2 shows the reliability and internal consistency of the questionnaire used, the consistency of the 11 subscales, and their average scores. The complete questionnaire showed a high-reliability index (α = 0.944) for the total sample. All the subscales showed α values above 0.790, except subscale 11 (Health Pressures). Analyzing the results for the whole sample (N = 917), the subscales with the highest scores were No. 3 (Ill-Health Avoidance and Positive Health) followed by No. 2, 6, and 10 (Revitalization and Enjoyment, Strength and Endurance, and Challenge).

The score of each subscale was measured according to the gender of the participants. Of all the 11 subscales of the questionnaire, punctuations in girls showed higher scores than in boys, specifically in subscales 1, 3, 8, 9, and 11. In the rest of the questionnaire’s subscales, boys presented higher scores than girls in the practice of PE. The highest scores for boys were obtained in subscale 6. Subscale 3 had the highest score for girls and the second highest for boys, with almost the same results. The second highest-scored subscale for girls was number 10. Significant differences were observed in subscales 2, 4, 5, 6, 7, 9, and 10. The motives with the highest scores for boys were subscales 6, 3, 2, and 10, whereas girls’ motivations for exercising were subscales 3, 10, 9, and 2 (see Table 3).

Analyzing the subscales about the parameters of body composition (BMI, FAT %, and waist circumference), associations between the values of FAT % and BMI are observable. According to the average value established by Ortega et al. (2011) [34], with increasing values of these parameters, the school students attribute more importance to subscale 1 (Weight Management and Appearance) and less importance to subscale 2 (Revitalization and Enjoyment) (see Table 4).

**Figure 1:**
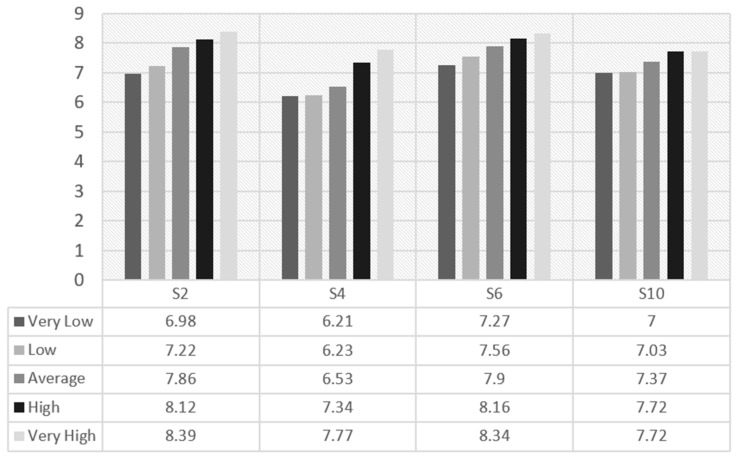
S2 (Revitalization and Enjoyment), S4 (Competition), S6 (Strength and Endurance), and S10 (Challenge) subscales scoring by cardiorespiratory fitness in boys.

Regarding possible associations between CRF and the different subscales, it was observed that boys’ scores in subscales Revitalization and Enjoyment, Competition, Strength and Endurance, and Challenge rose when the CRF level was increased (see Figure 1). Values are shown according to the average value established by Ortega et al. (2011) [34].

**Figure 2:**
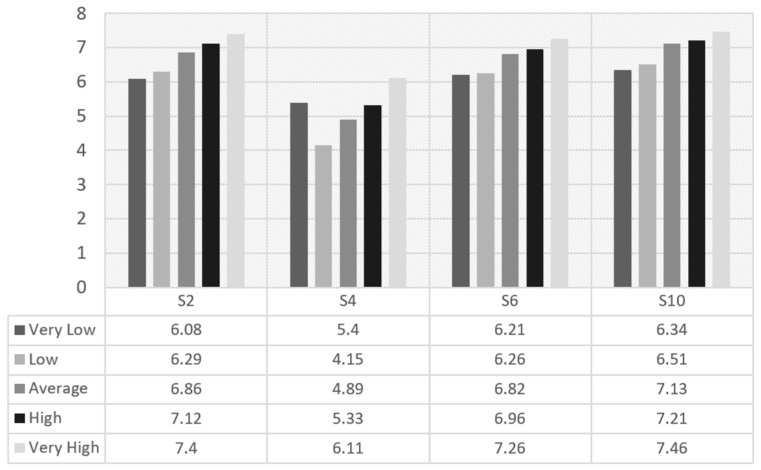
S2 (Revitalization and Enjoyment), S4 (Competition), S6 (Strength and Endurance) and S10 (Challenge) scoring by Cardiorespiratory Fitness in girls.

In the case of female participants and associations between the different subscales and the CRF level, the scoring related to subscales Revitalization and Enjoyment, Strength and Endurance, and Challenge increased when the CRF level was incremented (see Figure 2). Values are shown according to the average value established by Ortega et al. (2011) [34].

## 4. Discussion

It is argued that regular practice of PE is a fundamental tool available to the adolescent population to develop and improve their health [35]. Motivation, the process that stimulates and directs behavior toward the objective, is crucial in initiating and consolidating physical exercise [36]. In our research, the participants’ body composition was analyzed, and significant differences by gender were found. Differences in height, weight, fat percentage, and waist circumference were obtained. These results are similar to those from different studies in the adolescent population in which the girls presented higher levels of adiposity [37,38]. At the same time, the boys showed higher values in weight, height, and waist perimeter [39,40].

It is essential to clarify that, despite significant differences found in the variables mentioned above, both boys and girls present medium BMI values, body fat percentage, and waist perimeter. When comparing the weight of the population analyzed, similarity with the reference values stated by Ortega et al. (2011) was observed [34], in which the weight, height, and BMI in children, teenagers, and adults were transversally valued. In contrast to what Moreno et al. (2007) found, there was no prevalence of obesity amongst the students, considering that their values of body FAT %, BMI, and waist circumference are considered average [41]. Referring to the performance in the CRF test, this research shows significant differences (see Table 1) between boys and girls, with higher performance in boys. These results are aligned with previous studies with similar samples [42,43,44].

It is argued that the practice of PE during adolescence should be maintained over time, according to the scientific research associated with this issue [1,45]. However, a high drop-out rate is expected at this age stage [46,47]. Due to this reason, it is essential to know the reasons that lead adolescents to practice PE. The present study analyzed the motives for practicing PE in adolescents and the possible associations with the parameters of body composition and CRF. Descriptive analysis of the different subscales shows that participants, regardless of gender, engage in PE for Ill-Health Avoidance and Positive Health, Revitalization and Enjoyment, Strength and Endurance, and Challenge motives. These results are aligned with those found by similar projects [48,49,50].

Moreover, our research is aligned with several studies that highlighted well-being and fun as the most relevant incentives when exercising [51,52]. Thus, our results are aligned with the AVENA study [49], which shows that participants show a greater orientation towards attitudes related to physical sports activities. The current study participants scored Revitalization and Enjoyment, Challenge and Strength, and Endurance higher than the other subscales. Other studies [53] suggest that perceived competition is a clear motive for exercising. Our results are partially consistent with that, as the Competition subscale shows a positive score for the boys but not for the girls, which is in line with previous findings [54]. 

Concerning gender differences in the motives for practicing PE, our study agrees with a recent study [55] which concluded that intrinsic reasons influenced sustained exercise in adolescents. This research shows almost identical scores for boys and girls in the Weight Management and Appearance subscale. Considering the traditional attribution of this subscale to females, it suggests boys have a growing interest in aesthetics and body image [56]. Several authors recognize this trend as they have found that adolescents (both boys and girls) give more and more importance to their body image [37,57,58,59]. These results differ from those obtained by Moreno et al. (2013) [58], who concluded that weight and physical appearance were valued to a greater extent by female participants. The influence of the present aesthetic and beauty canon possibly justifies the equality of scoring among boys and girls, which is supported by the study of Wilson and Rogers [60]. The practice of physical exercise for reasons of improving physical appearance and appearance in both genders could also be motivated by the high prevalence of physical inactivity [6,7,15] in the Spanish adolescent population, which would result in higher levels of adolescent overweight and obesity [10,12], and might provide justification for the change in trend. 

Concerning the associations between the body composition parameters and the motives for PE practice, our study agrees with findings shown by several studies [61,62,63,64], which state that higher BMI and FAT % predicted greater exercise motivation for Weight Management and Appearance. Associations between CRF and motives for PE performance have been obtained in the present study. These findings are supported by the conclusions of other research conducted on the adolescent population. It also demonstrates that moderate to vigorous physical fitness is associated with sports competence (Challenge and Competition) and strength competence (Strength and Endurance) [65].

### 4.1. Implications for Health Policy, Practice, and Equity

The outcomes of this research provide important implications for understanding what makes adolescents engage in PE, the practice of PE from an early age, and how to reduce future obesity. For this reason, the present research argues for public policies which improve the younger population’s health. Thus, the role of physical education educators and caretakers necessarily includes promoting motivational strategies that foster intrinsic motivation toward physical exercise among adolescents. The younger population should be encouraged to exercise (taking advantage of their competitive impulse), to become more physically active, and, consequently, to promote a positive perception of their body image throughout their lifespan. This study also encourages physical education educators to develop exercise programs focused on positive changes in body composition parameters, increasing cardiorespiratory fitness levels, strength, and endurance.

### 4.2. Limitations

The data obtained in the current research need to be considered cautiously, since the sample considered is specifically contextualized and, considering the cross-sectional model of this study, causality relationships are difficult to establish. Notwithstanding all limitations, the results obtained in the research are worthy and accurate in adding knowledge about the motivations of PE in association with variables of body composition and cardiorespiratory fitness among adolescents. Future research should take into consideration other influential psychosocial variables in the performance of PE by adolescents, as well as the design of an experimental study that would help to discover causal relationships between the variables analyzed.

## 5. Conclusions

This research aimed to analyze the motives for practicing PE in adolescents and the possible associations with the parameters of body composition and CRF. Our participants highlighted the main reasons for participating in PE as avoiding potential health problems and improving their current health status (Ill-Health Avoidance and Positive Health subscale). Moreover, the boys scored the highest in subscale 6 (Strength and Endurance), which means that PE was practiced by them to improve the muscular component of physical fitness: more Strength and Endurance. Girls scored high in subscale 10 (Challenge), which implies that they understand the practice of PE as a tool to achieve short-term objectives: keeping an active lifestyle becomes a challenge to overcome. Due to all the reasons mentioned above, it is concluded that the motives that encourage participants in this research to engage in PE are purely intrinsic. 

It should be noted that this research shows a changing trend in terms of physical appearance and body image as the boys noted their concern about it. In addition, if the attitude or willingness toward PE is primarily based on appearance, it may be connected to the relationship between eating and body image [66,67]. The least significant motives of the participants for practicing PE were Stress Management, Social Recognition, and Health Pressures. That is the reason PE based on extrinsic motives is rejected in this study. Therefore, it is essential that educators and caregivers properly supervise the motives which encourage adolescents to engage in PE.

## Figures and Tables

**Table 1:** Anthropometric characteristics and endurance parameters (N = 917).

	Total	Gender		
	(N = 917)	Boys (n = 458)	Girls (n = 459)	
Variables	M ± SD	M ± SD	M ± SD	*p*-Value
Age (years)	14.83 ± 1.10	14.83 ± 1.11	14.83 ± 1.10	0.883
Weight (kg)	56.75 ± 13.48	59.00 ± 14.69	54.49 ± 11.73	<0.001 *
Height (m)	1.67 ± 0.10	1.65 ± 0.11	1.61 ± 0.49	<0.001 *
BMI (kg/m^2^)	21.67 ± 4.53	21.45 ± 4.16	21.89 ± 4.86	0.141
Body FAT (%)	22.36 ± 8.49	17.82 ± 7.76	26.88 ± 6.55	<0.001 *
Waist (cm)	72.01 ± 10.16	74.34 ± 10.84	69.68 ± 8.84	<0.001 *
Endurance (CRF)	5.15 ± 2.16	6.09 ± 2.37	4.22 ± 1.40	<0.001 *

Note: SD = Standard Deviation, BMI = Body Mass Index, Waist = Waist Circumference (* *p* < 0.01). CRF = Cardiorespiratory Fitness.

**Table 2:** Internal consistency and total score of AMPEF and its 11 subscales (N = 917).

Subscales	Cronbach Alpha	Mean (SD)
S1. Weight Management and Appearance	0.879	6.38 (2.54)
S2. Revitalization and Enjoyment	0.904	7.16 (2.46)
S3. Ill-Health Avoidance and Positive Health	0.812	7.64 (1.93)
S4. Competition	0.887	5.75 (3.14)
S5. Affiliation	0.794	6.13 (2.62)
S6. Strength and Endurance	0.847	7.12 (2.34)
S7. Social Recognition	0.790	4.43 (2.67)
S8. Stress Management	0.807	5.58 (2.87)
S9. Nimbleness	0.809	6.61 (2.64)
S10. Challenge	0.811	7.03 (2.34)
S11. Health Pressures	0.617	3.09 (2.71)
Total (48 Items)	0.944	6.27 (1.68)

**Table 3:** AMPEF results by gender-related subscales (N = 917).

Subscales	Boys (n = 458. M ± SD)	Girls (n = 459. M ± SD)	*p*-Value
S1. Weight Management and Appearance	6.40 ± 2.41	6.46 ± 2.67	0.116
S2. Revitalization and Enjoyment	7.60 ± 2.21	6.72 ± 2.61	0.000 *
S3. Ill-Health Avoidance and Positive Health	7.63 ± 1.85	7.65 ± 2.01	0.487
S4. Competition	6.57 ± 2.85	4.92 ± 3.21	0.000 *
S5. Affiliation	6.54 ± 2.47	5.72 ± 2.70	0.000 *
S6. Strength and Endurance	7.65 ± 2.16	6.60 ± 2.40	0.000 *
S7. Social Recognition	4.89 ± 2.70	3.97 ± 2.57	0.000 *
S8. Stress Management	5.46 ± 2.92	5.70 ± 2.82	0.267
S9. Nimbleness	6.42 ± 2.68	6.80 ± 2.82	0.027 *
S10. Challenge	7.18 ± 2.27	6.87 ± 2.39	0.040 *
S11. Health Pressures	3.08 ± 2.69	3.10 ± 2.74	0.968

Note: M = Mean, SD = Standard Deviation (* *p* < 0.05).

**Table 4:** S1 (Weight Management and Appearance) and S2 (Revitalization and Enjoyment) subscales scored by FAT % and BMI.

S1. Weight Management and Appearance										
FAT %	Very Low		Low		Average		High		Very High	
	Boys	Girls	Boys	Girls	Boys	Girls	Boys	Girls	Boys	Girls
S1	5.79	4.97	5.86	5.42	6.49	6.64	6.51	6.96	6.51	6.46
S2	8.11	6.93	8.07	6.84	7.69	6.80	7.05	6.71	6.56	6.65
**S2. Revitalization and Enjoyment**										
**BMI**	**Very Low**		**Low**		**Average**		**High**		**Very High**	
	**Boys**	**Girls**	**Boys**	**Girls**	**Boys**	**Girls**	**Boys**	**Girls**	**Boys**	**Girls**
S1	5.50	5.36	5.79	6.26	6.54	6.64	6.59	7.04	6.91	6.58
S2	7.91	6.79	7.78	7.17	7.70	6.75	7.53	6.93	6.83	6.44

## Data Availability

Data originated during the research project is available on request from the corresponding author (igarcia@uloyola.es).
